# The Aspects of Artificial Intelligence in Different Phases of the Food Value and Supply Chain

**DOI:** 10.3390/foods12081654

**Published:** 2023-04-15

**Authors:** Vaida Bačiulienė, Yuriy Bilan, Valentinas Navickas, Lubomir Civín

**Affiliations:** 1 School of Economics and Business, Kaunas University of Technology, 44249 Kaunas, Lithuania; vaida.baciuliene@ktu.edu (V.B.); valna@ktu.lt (V.N.); 2 Faculty of Economics and Management, Czech University of Life Sciences, 16500 Prague, Czech Republic; civinl@pef.czu.cz; 3 Lithuania Business University of Applied Sciences, 91249 Klaipeda, Lithuania

**Keywords:** artificial intelligence, food supply chain, artificial intelligence challenges

## Abstract

The types of artificial intelligence, artificial intelligence integration to the food value and supply chain, other technologies embedded with artificial intelligence, artificial intelligence adoption barriers in the food value and supply chain, and solutions to overcome these barriers were analyzed by the authors. It was demonstrated by the analysis that artificial intelligence can be integrated vertically into the entire food supply and value chain, owing to its wide range of functions. Different phases of the chain are affected by developed technologies such as robotics, drones, and smart machines. Different capabilities are provided for different phases by the interaction of artificial intelligence with other technologies such as big data mining, machine learning, the Internet of services, agribots, industrial robots, sensors and drones, digital platforms, driverless vehicles and machinery, and nanotechnology, as revealed by a systematic literature analysis. However, the application of artificial intelligence is hindered by social, technological, and economic barriers. These barriers can be overcome by developing the financial and digital literacy of farmers and by disseminating good practices among the participants of the food supply and value chain.

## 1. Introduction

In this study, the aspects of artificial intelligence use in the food value and supply chain will be comprehensively evaluated. The concept of vertical integration in the food industry and how AI can be integrated into different stages of the food value and supply chain will be explored, along with the identification of key barriers to its application. By providing a retrospective evaluation of previous publications on this topic, new contributions will be identified, research trends captured, and prospective areas for future investigation highlighted.

The need for this study stems from the fact that the agrifood industry plays a crucial role in the country’s development and affects the global economy. However, key participants in the food supply and value chain are forced to seek new solutions and competitive advantages due to changing competitive environments. The food supply chain is complex, and previous research has focused on various phases of the food system separately [1,2,3,4,5]. However, there is a lack of research on the use of artificial intelligence (AI) in the food supply chain as a whole [6,7,8]. To address this gap in knowledge, this study aims to evaluate the use of AI in all stages of the food system.

The authors argue that the increasing body of knowledge related to AI requires a new approach to comprehend the knowledge structure of this field. Therefore, this study will provide a retrospective evaluation of the publications accumulated to reveal new contributions, capture research trends, and identify prospective areas for future investigation. The study aims to answer the following research questions:

- What are the types of artificial intelligence?
- How can artificial intelligence be integrated into the food and value supply chain’s different phases?
- What are other technologies connected with artificial intelligence?
- What are the key barriers to AI application in the food and value supply chain?

By addressing these questions, the current understanding of the role of AI in the food supply chain will be contributed to, and opportunities for future research and development in this field will be identified.

## 2. Methodology

The empirical approach used in this study is a systematic literature review, which is a widely recognized method in academic research to identify and analyze relevant literature on a specific topic. Peer-reviewed research articles were collected from the Internet through Google Scholar and Science Direct using search terms related to artificial intelligence systems and their applications in the food supply chain. To determine the relevance of the articles, a multistep screening process was employed. Initially, 400 papers were downloaded from the Internet based on their titles. After removing 50 duplicates, 350 articles were assessed based on their abstracts. Out of the remaining 245, 190 articles were removed based on their content, leaving 55 articles that were used for this review. Various tests were not used in this study since it is a literature review that focuses on synthesizing and analyzing existing knowledge and research on the use of artificial intelligence in the food supply chain. However, the selected articles were thoroughly analyzed to identify the key themes, trends, and research gaps related to the use of AI in the food supply chain. The articles were also evaluated based on their research quality, rigor, and relevance to the research objectives of this paper. The systematic literature review method was chosen because it is a robust and objective approach to analyzing the existing body of knowledge on a specific topic. It also allows for a comprehensive assessment of the research gaps and opportunities in the field of AI and the food supply chain.

## 3. Literature Review

### 3.1. The Concept of Artificial Intelligence

As artificial intelligence continues to evolve, the concept of AI is also constantly changing. The scientific literature has identified different stages of AI evolution, including artificial narrow intelligence, artificial general intelligence, and artificial superintelligence [1,9,10,11]. Artificial narrow intelligence is designed to perform specific tasks or operate within limited areas. In contrast, artificial general intelligence is capable of solving complex problems and is described as a neutral intelligence that can autonomously control itself [12,13]. This type of AI should be able to adapt to various environments, solve different problems, and make connections between seemingly unrelated fields of application [13]. Artificial superintelligence, would render humans redundant due to its self-awareness, scientific creativity, social skills, and general wisdom [14].

**Figure 1:**
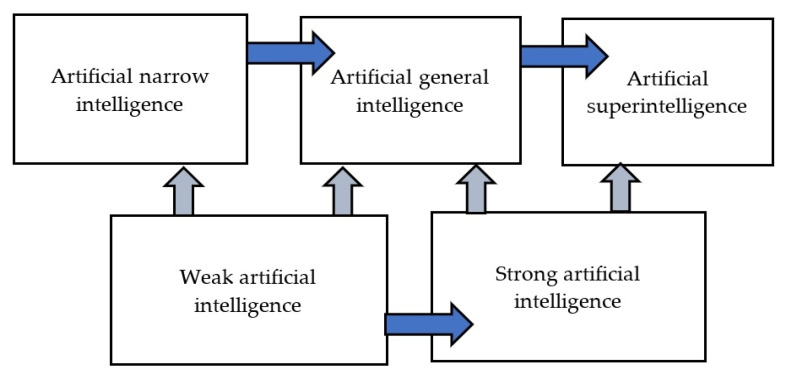
Stages in the evolution of artificial intelligence (own).

AI can be classified as weak or strong based on its stage of evolution. Weak AI is equivalent to artificial narrow and general intelligence [10,14], while strong AI is a more ambitious version of AI. Fjelland (2020) notes that although general artificial intelligence is classified as weak AI, it is a step closer to strong AI and can also be classified as such [10] (see Figure 1).

“Artificial intelligence” (AI) term is often used with diverse meanings across various scientific disciplines [15]. Current scholarly debate in technical contexts navigates between limited and domain-specific forms of “narrow intelligence”, more sophisticated forms of human-like “general AI”, and “super AI” that go significantly beyond human cognitive abilities [16], while its meanings go more on to casual aspects [17]. The range of AI debate is extending with process automation concepts [18].

Although there are not many classifications of artificial intelligence in the scientific literature on the continuous development of the technology concept (see Table 1), Haenlein and Kaplan (2019) [19] based on the stages of artificial intelligence evolution, distinguished types of artificial intelligence systems: (1) analytical artificial intelligence creates a cognitive representation of the world and uses learning to make decisions based on past experience. Most artificial intelligence systems used by businesses fall into this category, such as image recognition and autonomous vehicles; (2) human-inspired intelligence has both cognitive and emotional intelligence elements. In addition to depicting the cognitive world, these systems are able to understand and take human emotions into account in decision making, such as intelligent visual systems that recognize emotions such as anger or joy used by companies to recognize emotions when interacting with customers or hiring new employees; (3) humanized intelligence combines cognitive, emotional, and social intelligence competencies, but there are no such systems that are conscious and perceived in interaction with others; only prototype tests are performed.

Davenport et al. (2020) [4] classify artificial intelligence by type of task and by embeddedness. According to the type of task, artificial intelligence is classified into (1) analyzing numbers and (2) analyzing digital data (e.g., text, voice, images, or facial expressions). Although artificial intelligence requires input before decisions can be made, analyzing numbers is simpler than numerical data. In terms of embeddedness, artificial intelligence is divided into (1) virtual forms (e.g., artificial intelligence used in smartphones and digital platforms) and (2) real forms (e.g., robots). At the same time, Davenport et al. (2020) [4] point out those virtual and real forms of artificial intelligence should not be understood as separate categories but as a continuum in which artificial intelligence objects exist.

Strusani and Houngbonon (2019) [2] classify artificial intelligence according to the simulation and automation of cognitive abilities as (1) basic, (2) advanced, and (3) autonomous. Basic artificial intelligence inserts cognitive abilities (memory, language, attention, etc.) and makes decisions. This type of artificial intelligence is most commonly used in business to improve analytical decisions or the performance of digital platforms. Advanced artificial intelligence models include cognitive abilities, such as perception, sight, and perception of space. This type of artificial intelligence is able to analyze unstructured data (text, image, sound, etc.). It is expected that independent artificial intelligence will be able to communicate with people and learn independently, but it is not yet widely used, and only prototypes of this type are being developed, e.g., Fetch Robotics, Boston Dynamics, Hanson Robotics [2].

Artificial intelligence can be classified according to the functions of applications into (1) descriptive, (2) predictive, and prescriptive [3,5]. Descriptive artificial intelligence extracts data using sensors and reveals new connections. Predictive artificial intelligence can operate in an area where there are clear cause-and-effect relationships. Prescriptive artificial intelligence creates guidelines for actions to be taken [5].

From an economic perspective, technologies based on artificial intelligence are becoming a strategic business interest. Artificial intelligence is defined as a technical advantage that cannot be replicated by other technologies. A study by McKinsey (2018) [20] analyzing 400 use cases in 19 sectors and 9 business functions found that for 69 percent, artificial intelligence improved traditional analysis methods. The competitive advantage of artificial intelligence over primary technological principles (modeling, statistics, heuristics, and other classical optimizations) is identified as a specifically broad range of functions that can be widely applied in business because it is objective intelligence that solves uncertain, complex situations and generates new solutions [21]. Artificial intelligence as a competitive advantage due to its increasing use will not be gained simply by applying it in the food supply chain. In the future, ways will need to be explored to apply artificial intelligence creatively to create a positive competitive impact. Artificial intelligence technologies inevitably transform the agricultural sector as well, affecting all phases of the food supply chain.

### 3.2. Vertical Integration of Artificial Intelligence in the Food Supply Chain

The food industry is not a simple traditional sector but rather a complex business network that operates in the value chain from production to consumption. To stay competitive and sustainable, the industry must continuously evolve with the latest technology. Artificial intelligence (AI) can help increase the productivity of the food industry. Machine learning allows us to predict crop yields, determine irrigation intensity, and sense soil content. AI can also be used for video surveillance and robots to monitor crops, detect weeds, and automate weed and plant sowing and planting [22]. AI can be used for the early detection of plant diseases, creating detailed soil maps for damage control, fertilization, and controlling water and energy consumption. Animal welfare can be assessed, and milking can be performed robotically [23,24,25,26]. Technological solutions based on AI enable more products to be produced at lower costs and increase supply chain efficiency while improving product quality and safety and ensuring faster time-to-market [27,28,29,30]. As advanced countries face long-term labor shortages in agriculture, AI and ML smart vehicles, agribots, drones, and machine learning robotics are reasonable options for many agricultural operations [31]. At different stages of the food value and supply chain, artificial intelligence provides different empowerments using a combination of different technologies (see Table 2 and Table 3). The integration of artificial intelligence with connected and embedded technologies in the food value and supply chain enables the transformation of agrifood chain processes, replacing them with more efficient methods to increase productivity in the agrifood industry. Below are the ways in which AI and other technologies can be integrated into agrifood supply and value chain.

**Figure 2:**
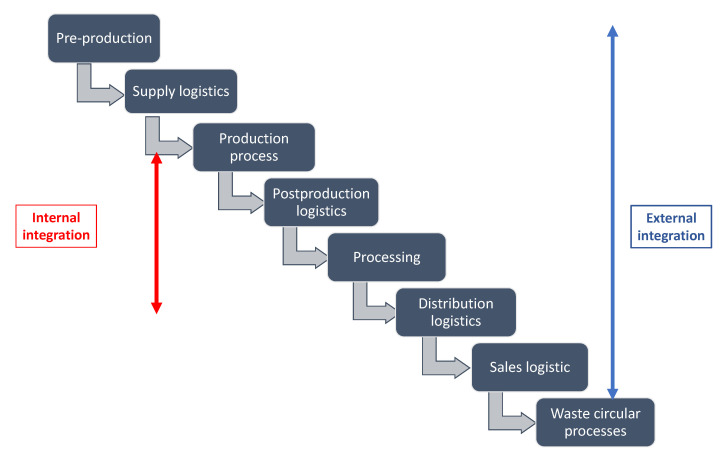
AI vertical integration of food value and supply chain (own).

Vertical integration of AI technology in the food supply chain is possible when there is traceability in all parts of the chain and information and data are shared between the participants. This integration can be divided into internal and external (see Figure 2).

Vertical internal integration takes place through the application of artificial intelligence in the supply chain, which consists of four main stages—preproduction, manufacturing, processing, and distribution [32]. In the preproduction phase, artificial intelligence becomes a tool for determining soil properties, preparing previous crops for new sowing, and controlling irrigation systems. An agricultural technique using ML algorithms to identify real-time data sets from drones and understand patterns in large-scale data by yield mapping can improve crop yield prediction, an invaluable technology for crop planning [31,33]. In the agricultural production phase, the applicability of artificial intelligence is wider—weather forecasting helps to harvest in time, weed detection helps to preserve more abundant yields, optimize the appropriate mix of biodegradable pesticides in their application volume, optimally use bioenergy, regulate nutrition and water content according to the individual animal and vegetation needs, monitor animal health, monitor crops, and process data—it all reduces costs and improves agricultural production efficiency.

The specifics of the comprehensive food value and supply chain can be defined in a more detailed way, e.g., eight phases including different circular economy processes and environmental protection elements. (see Figure 2.) Moreover, vertical external integration of agriculture into the food value chain is the next step, following the output of raw materials into the foodstuff industry. It is more similar to specific models in Industry 4.0. Mainly it deals with changes in processing processes known today into the new ones represented by the concept of the smart factory. The change consists of transforming the traditional structure determined by the distinction between information and operation technologies into a model where physical processes monitored by cyber-physical systems create their virtual representation and, supported by ML, are involved in decision making processes. Such systems enable new ways of communication and cooperation among devices, production assets, and information systems, which definitely ensure product quality. In the processing and sales phases, AI is also used for marketing purposes to analyze demand and manage production volumes.

Precise forecasting for crop prices based on predictable yield rates allows for predicting total harvest volumes while supporting pricing strategies for a given crop. In the distribution and sales logistics phases of supply chains, AI and IoT applications with drones promote track and traceability of transportation means contributing to removing or avoiding road traffic jams and supporting quality, safe, and fresh foodstuff supplies to markets [34].

Within the concept of Industry 4.0, one of the critical functions for integrating individual elements into an integrated complex, even for food value and supply chains, is the development of platforms. Industry 4.0 concept implementation in agriculture and food processing will depend on fundamental technological advances implemented to new business models extensively utilizing AI and the entire complex of its components represented by the digitalization process. The dominant role in the future is the challenge of implementing digital manufacturing platforms. Digital platforms for manufacturing play a crucial role in the entire value and supply chain’s competitiveness. Their role will be to integrate vertically new technologies, apps, and services into one technology by a complex of interlinked data and processes [35].

Vertical external integration of artificial intelligence includes integration within the internal integration but includes external participants: suppliers, retailers, and end users (consumers). Artificial intelligence technologies provide an opportunity to study the properties of seeds and analyze data in laboratories. In the harvest processing phase, innovative food from primary agricultural products is created with the help of artificial intelligence, and packaging processes are carried out robotically [36]. In the retail and logistics phase, autonomous stores can be set up using artificial intelligence; artificial intelligence algorithms are used to analyze consumer data to manage sales in e-shops. In the last phase of the food supply chain, data related to consumers and their habits are analyzed [37,38].

The purposeful use of artificial intelligence requires the use of Big Data mining technologies. A lot of data is generated in the food supply chain—soil properties, climate, spatial, moisture, animal health, and economic data. By analyzing this data with artificial intelligence with Big Data mining algorithms, the resulting insights help make better decisions, manage the food supply chain, ensure supply/demand, and reduce food supply chain fluctuations and delays [32,39].

**Table 3:** Food value and supply chain phases linkage to AI components (own on basis of [29,39,40,41,42]).

Value & Supply Chain Phase	AI Components Embedded (for Abbreviations of Technology See Table 2)										
-	S&D	BD	ML	IoT	IoS	AGR	DVM	IR	NT	3DP	DP
**Preproduction phase**											
Climate change & natural processes control	x	x	x		x						
Weather forecasts and control	x	x	x								
Irrigation management	x	x		x	x	x	x				
Waste recirculation control	x		x		x		x				x
Crop yield assessment	x	x	x								
**Supply logistics phase**											
Control of input inventory	x	x	x		x		x				x
Orders and logistics			x		x						x
Finance management	x		x		x						x
On spot distribution					x	x	x				
**Agricultural production phase**											
Phyto- and livestock nutrition supervision	x	x	x	x							x
Nutrition management	x	x	x		x	x			x		x
Disease prevention, detection & treatment	x	x	x	x	x				x		
Livestock & crop production management	x	x	x		x	x	x				x
Agritechnics & equipment servicing	x			x	x						x
**Postproduction logistics**											
Crops storage	x	x	x	x	x	x					x
Transport to processing facilities					x		x				x
Financing	x		x		x						x
**Food processing phase**											
Demand management		x	x			x			x		
Production management	X		x	x	x			x		x	x
Quality management	x							x			
Distribution logistics				x	x		x				x
**Sales phase**											
Inventory control	x	x	x								
Storage	x	x	x	x	x	x					x
Trade	x	x	x	x	x		x			x	x
Consumer analysis	x	x	x	x							x
**Waste recirculation**											
Waste collection and sorting	x	x		x		x	x				
Waste processing	x	x	x	x			x			x	x
Processed waste storage	x	x		x							x

At present, consumers are particularly demanding, wanting to be informed about farm markets, production practices, and marketing strategies, leading to the introduction of additional regulations and market-oriented standards in the development of food supply chains [40]. The European Commission of 2019 presented the Green Deal strategy. The strategy aims to make the European Union a modern, resource-efficient society with a competitive economy. The Farm to Fork program is part of the Green Deal strategy, which will commit to reducing the environmental footprint and climate change impacts. The food supply chain will have to be environmentally neutral, protect the health and welfare of land, water, plants, and animals, and promote organic production on farms [41].

All stages of food production and transportation due to high standards, restrictive government regulation, perishable products, and limited stockpiling to meet demand need to be coordinated in a particularly coherent way, leading to food supply chain processes such as production, stockpiling, logistics, seen as a multilevel system. The analysis of Violi (2020) [40] and Pournader et al. (2021) [42] revealed that artificial intelligence tools are used for a variety of supply chain operations to facilitate decision making. Artificial intelligence methods are widely used for inventory management, demand forecasting, and risk management. In order to gain a competitive advantage, food supply chain key participants need to develop strategies for inter-organizational processes that focus on artificial intelligence. Sharing information between organizations can reduce the negative impact of an ever changing business environment.

### 3.3. Artificial Intelligence Adoption for Risk Management in Food Value and Supply Chains

Various studies [43,44] show that the use of artificial intelligence systems in the food value supply chain is uneven, with each stage of the supply chain (preproduction, production, premarketing and transport, transport and storage, process and production, retail, consumption, and waste) differing in technology susceptibility. Uneven integration and adaptation of artificial intelligence complicate digital transformation, maximizing efficiency, profit, and productivity gains. It is important to be able to apply artificial intelligence technologies to target capital technology according to the susceptibility of the phases of the food value supply chain in order to have an even distribution of artificial intelligence technology throughout the food value supply chain.

The food value supply chain describes activities related to the production and distribution of agricultural or horticultural products from farm to fork [45]. In this chain, farmers, distribution, processing companies, and marketing experts must work together to deliver food to consumers to meet their needs at the highest level of service [46,47]. Food supply chain management requires solutions that optimize the number of resources involved in the sequential stages of production and logistics processes. The food supply chain also differs from other chains in the factors that affect it: food quality and safety and the variability of natural conditions [48,49].

The role of artificial intelligence in managing the factors affecting the food supply chain can be classified into two main segments: food quality management and food safety management. Artificial intelligence can regulate the amount of pesticides in food; increase the production of high-quality food. The use of robots in warehouses is also seen as a food quality management tool, as it reduces the likelihood of human error or violation of sanitary rules. Sensors and artificial intelligence algorithms are used to ensure food safety. The most popular product in this field is the electronic nose, which is used to assess the freshness of food. The amount of fertilizer is regulated in the preproduction phase by artificial intelligence. Image processing and recognition technologies help to assess the safety of products in several phases of the food supply chain—preproduction, production, process, and harvest processing.

The challenge for the food industry is to bring safe and high-quality products to markets to meet consumer needs. The main goal is to develop standard, reliable procedures for product quality control. However, the food industry tends to work with different, variable raw materials, and their processing is characterized by non-linear behavior. Market participants (sellers and buyers) have reached a consensus on how to assess the quality of a particular product. This consensus is supported by common unsystematic rules followed by experts. The work of experts is, in turn, based on experience. As this benefits market participants, the modern market needs to standardize quality assessment procedures based on subjective human behavior. However, the training of classifiers used in artificial intelligence is difficult and expensive because they have to gain a lot of experience before doing the work. The classifications of artificial intelligence need to be periodically reclassified to avoid significant individual differences in assessments. Despite the complexity of artificial intelligence training procedures, the repetition of assessment is low, which affects market confidence. As a result, the use of artificial intelligence techniques in the food industry that can make accurate and reproducible assessments is a difficult task [50].

### 3.4. Artificial Intelligence Adoption Barriers and Their Solutions in Food Value and Supply Chain

The use of artificial intelligence in the food supply chain can solve the challenges of food safety, quality, and fluctuations in the food supply chain but the opportunities for wider use are limited by barriers that hinder the development of this technology. Barriers to the application of artificial intelligence are usually categorized into social, technological, and economic barriers (see Table 4).

Researcher Cubric (2020) [54] also divided barriers to artificial intelligence adoption into three categories: economic barriers (costs, support infrastructure), technical barriers (data access, digital infrastructure), and social barriers (robot dependency, job retention, lack of knowledge, and security). Lezoche et al. (2020) [51] distinguished barriers to artificial intelligence adoption into social (person replacement with artificial intelligence is considered a threat), technological (artificial intelligence must be developed to facilitate the development of visualization, integration of language interfaces into systems can benefit users in remote areas, no artificial intelligence expert system able to create creative responses to what people can do in unexpected situations, lack of flexibility and little opportunity to adapt to a changing environment), and economic barriers. Bičkauskė and others (2020) [52] identified cyber security as one of the key challenges, which particularly hampers the use of artificial intelligence, leading to mistrust of technology. Liu et al. (2021) [53] emphasize three aspects that hinder the development of artificial intelligence: it is still difficult to find standardized technological solutions, the gap between artificial intelligence users, and artificial intelligence developers require a large amount of data to train artificial intelligence models.

Barriers to artificial intelligence can be removed or reduced by developing financial and digital literacy among farmers. The low level of entrepreneurship of farmers and the conservative approach to farming as a business lead to the search for proven artificial intelligence solutions for the farm, therefore the dissemination of good practices and faster development of artificial intelligence technology, standardizing solutions, would help to overcome barriers to artificial intelligence [55].

## 4. Conclusions

Artificial intelligence is rapidly evolving, which leads to constantly changing concepts of artificial intelligence. The evolution of artificial intelligence has become the basis for classification. According to the stages of evolution, artificial intelligence is classified into analytical, human-inspired, and humanized. Artificial intelligence is also classified by task type (analyzing numbers or numerical data), by embedding (virtual or real forms), by simulating and automating cognitive abilities (basic, advanced, autonomous), and by application functions (descriptive, predictive, prescriptive). The types of artificial intelligence, in the continuous evolution of technology, may also change in the future.

The diverse range of functions that AI offers can be integrated vertically into the entire food supply and value chain. A systematic literature analysis revealed that technologies like robotics, drones, and smart machines, which interact with AI and other technologies (such as Big Data Mining, Machine Learning, Internet of Services, Agribots, Industrial Robots, Sensors and Drone, Digital Platforms, Driverless Vehicles and Machinery, and Nanotechnology), provide different capabilities for different phases of the chain. However, the successful application of AI is impeded by social, technological, and economic barriers. To overcome these obstacles, it is necessary to develop financial and digital literacy and disseminate good practices among the participants in the food supply and value chain.

## Figures and Tables

**Table 1:** Classifications of artificial intelligence.

According to the Stages of Evolution of Artificial Intelligence [19]	By Task Type [4]	By Embeddedness [4]	According to the Simulation and Automation of Cognitive Abilities [2]	According to the Functions of Applications [3,5]
Analytical Human-inspired Humanized	Analyzing numbers Analyzing digital data	Virtual form Real form	Basic Advanced Autonomous	Descriptive Predictive Prescriptive

**Table 2:** Artificial Intelligence connected and embedded technologies.

Big Data Mining	BD
Machine learning	ML
Internet of Services	IoS
Internet of Things	IoT
Agribots	AGR
Industrial Robots	IR
Sensors and Drones	S&D
Digital Platforms	DP
Driverless Vehicles and Machinery	DVM
Nanotechnology	NT

**Table 4:** Barriers to artificial intelligence adoption (own, based on [15,51,52,53]).

Barriers to Artificial Intelligence Adoption	
**Social**	Replacing a person with artificial intelligence is considered a threat Data privacy and cyber security Lack of knowledge and education Distrust of technology The gap between users of artificial intelligence and developers of artificial intelligence
**Technological**	Artificial intelligence needs to be developed to facilitate the development of visualization Lack of language interfaces in the system Artificial intelligence expert systems are not capable of creating creative responses to what human experts can do in unexpected situations Lack of flexibility and low ability to adapt to changing environments Unable to recognize an object or situation where no response is installed Lack of standardized technological solutions Large amounts of data required
**Economic**	Lack of financial resources Lack of financial support infrastructure

## Data Availability

The datasets generated for this study are available on request to the corresponding author.
